# Utilizing biological experimental data and molecular dynamics for the classification of mutational hotspots through machine learning

**DOI:** 10.1093/bioadv/vbae125

**Published:** 2024-08-26

**Authors:** James G Davies, Georgina E Menzies

**Affiliations:** Molecular Bioscience Division, School of Biosciences, Cardiff University, Cardiff, CF10 3AX, United Kingdom; Molecular Bioscience Division, School of Biosciences, Cardiff University, Cardiff, CF10 3AX, United Kingdom

## Abstract

**Motivation:**

Benzo[*a*]pyrene, a notorious DNA-damaging carcinogen, belongs to the family of polycyclic aromatic hydrocarbons commonly found in tobacco smoke. Surprisingly, nucleotide excision repair (NER) machinery exhibits inefficiency in recognizing specific bulky DNA adducts including Benzo[*a*]pyrene Diol-Epoxide (BPDE), a Benzo[*a*]pyrene metabolite. While sequence context is emerging as the leading factor linking the inadequate NER response to BPDE adducts, the precise structural attributes governing these disparities remain inadequately understood. We therefore combined the domains of molecular dynamics and machine learning to conduct a comprehensive assessment of helical distortion caused by BPDE-Guanine adducts in multiple gene contexts. Specifically, we implemented a dual approach involving a random forest classification-based analysis and subsequent feature selection to identify precise topological features that may distinguish adduct sites of variable repair capacity. Our models were trained using helical data extracted from duplexes representing both BPDE hotspot and nonhotspot sites within the *TP53* gene, then applied to sites within *TP53*, *cII*, and *lacZ* genes.

**Results:**

We show our optimized model consistently achieved exceptional performance, with accuracy, precision, and f1 scores exceeding 91%. Our feature selection approach uncovered that discernible variance in regional base pair rotation played a pivotal role in informing the decisions of our model. Notably, these disparities were highly conserved among *TP53* and *lacZ* duplexes and appeared to be influenced by the regional GC content. As such, our findings suggest that there are indeed conserved topological features distinguishing hotspots and nonhotpot sites, highlighting regional GC content as a potential biomarker for mutation.

**Availability and implementation:**

Code for comparing machine learning classifiers and evaluating their performance is available at https://github.com/jdavies24/ML-Classifier-Comparison, and code for analysing DNA structure with Curves+ and Canal using Random Forest is available at https://github.com/jdavies24/ML-classification-of-DNA-trajectories.

## 1 Introduction

Mutations serve as the fundamental source of genetic variation, fostering adaptive evolution, whilst contributing to the development of diseases such as cancer and age-related illnesses (Alexandrov *et al.* 2013, Bae *et al.* 2018). A nucleotides mutation rate exhibits considerable heterogeneity throughout the genome, with the rate of site-to-site mutation shown to vary by >100-fold, prompting sequence dependant contribution to both evolution and genetic pathologies (Ellegren *et al.* 2003). Analysing the precise features that promote this dependency may unlock valuable insights into the mutagenic mechanisms underlying various human diseases.

It is widely accepted that site-specific mutational frequencies are contingent upon three generalized factors: (i) nucleotide stability and vulnerability to mutagenesis; (ii) the fidelity of DNA replication processes; and (iii) the efficiency of DNA repair machinery (Baer *et al.* 2007). Consequently, a natural gradient manifests, revealing the degree to which mutational occurrences are influenced by the underlying sequence composition. For instance, regions of repetitive DNA such as homonucleotide runs and microsatellite repeats serve as an active source of hypermutability through polymerase slippage events, emphasizing motif recurrence over precise sequence context (Jackson *et al.* 1998, Harfe and Jinks-Robertson 2000). In contrast, alternative mutation spectra exhibit pronounced sequence specificity, often guided by local, and occasionally distant, sequence contexts (Baylin *et al.* 1998, Page *et al.* 1998, Donny-Clark and Broyde 2009, Hodgkinson and Eyre-Walker 2011, Rogozin *et al.* 2018). While the underlying causes of the latter remain subjects of substantial debate, emerging evidence indicates that this interdependence forms the foundation for numerous disease-associated mutation spectra, notably that of the *TP53* mutation spectrum within the context of lung cancer (Hollstein *et al.* 1991, Toyooka *et al.* 2003).

*TP53* is a gene that is responsible for encoding the p53 tumour suppressor protein (Lane 1992). Its principal biological role involves safeguarding DNA integrity through the inhibition of cell proliferation and the promotion of apoptosis in response to cellular stress (Vogelstein *et al.* 2000). This renders *TP53* a pivotal target for inactivation in disease, and as such, establishes it as the most commonly mutated gene in human cancer (Hollstein *et al.* 1991). *TP53* mutations are particularly prevalent in lung cancer, with approximately 50% of tumours containing a mutation (Hainaut and Hollstein 2000). Mutation distribution, however, is shown to vary substantially between lung and other tumour types, with a higher frequency of G:C > T:A transversions in the former (Hollstein *et al.* 1991, Hainaut and Hollstein 2000, Béroud and Soussi 2003). While the influence of sequence context on the varying *TP53* mutation spectrum continues to be debated, a consensus exists regarding the underlying cause: the formation of DNA adducts induced by polycyclic aromatic hydrocarbons (PAH) found in cigarette smoke (Yu *et al.* 2002, Rodin and Rodin 2005, Xu *et al.* 2009).

**Figure 1.**
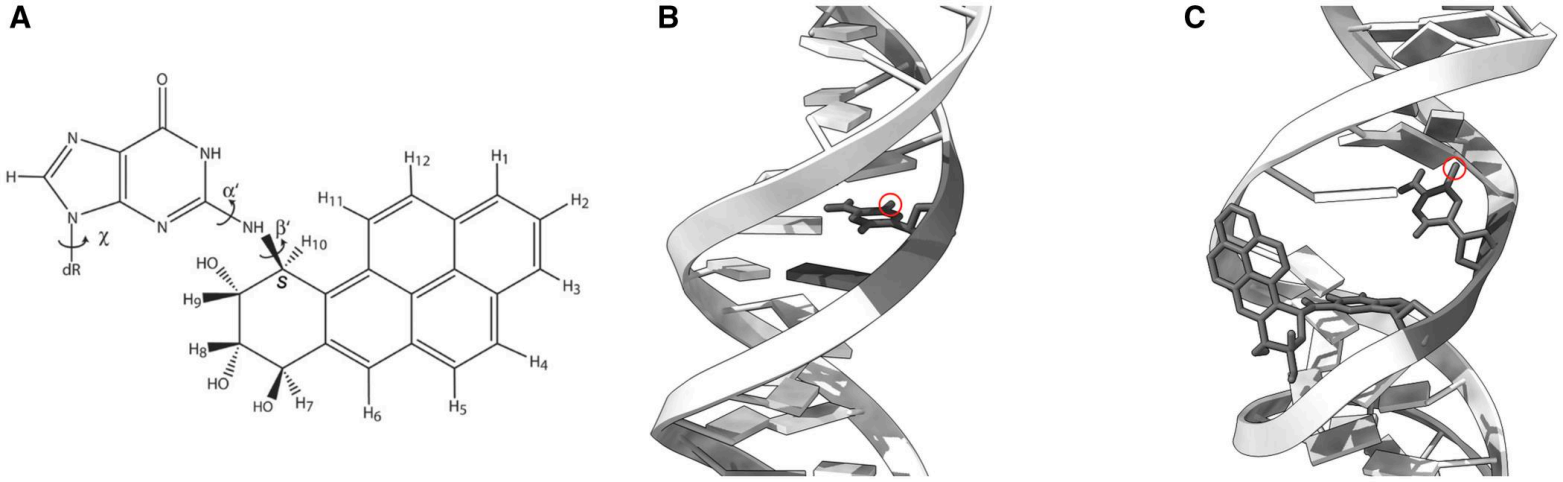
Chemical structure and mutagenic adduct formation of Benzo(a)pyrene diolepoxide. (A) Chemical structure of 7S, 8R, dihydroxy-9R, 10S-epoxy-7S, 8R, 9R, 10S (+)-trans-anti-B[*a*]PDE; (B) regular unadducted DNA with methyl group (highlighted by a circle) on the cytosine at base 6; (C) adduct of BPDE (sticks) bound to guanine in a CpG site in minor groove directed towards the 5′ end of the DNA sequence.

Benzo[*a*]pyrene (B[*a*]P) emerges as the predominant PAH formed from the incomplete combustion of organic material within tobacco smoke, with Vu *et al.*, establishing that one cigarette can yield a B[*a*]P intake of 13–38 ng (Vu *et al.* 2015). Subsequently, this compound is metabolically processed to generate a series of carcinogenic derivatives, including the trans(+)anti-benzo[a]pyrene diol epoxide (BPDE) (Fig. 1A) (Baird *et al.* 2005). Empirical evidence suggests that a combination of preferential DNA damage and poor repair is responsible for the lung associated mutation hotspots in *TP53* (Denissenko *et al.* 1996). Recurrently mutated codons 157, 158, 245, 248, 273, and 282 harbour methylated CG dinucleotides representing favourable locations for BPDE binding while concurrently exhibiting resistance to nucleotide excision repair (NER) mechanisms (Denissenko *et al.* 1998). Notably, the (K)-7S,8R,9R,10S+ anti-B[*a*]PDE enantiomer (10S) DNA adduct dominates, accounting for an overwhelming majority (70.8%–92.9%) of the total adducts formed at these specific sites (Fig. 1B and C) (Matter *et al.* 2004). Denissenko *et al.*, report other known targets of BPDE, including 170, 186, 202, 213, 267, and 290, whose mutation is either uncommon or silent in lung cancer (Denissenko *et al.* 1996, Chen *et al.* 1998, Yoon *et al.* 2003). Given the broad specificity of BPDE and accounting for selective processes, the induction of *TP53* mutations by BPDE is likely the combined result of differential adduct availability and varying repair capabilities at distinct adduct sites.

NER is capable of repairing a diverse range of bulky lesions, but its efficiency varies dramatically according to the chemical structure, stereochemistry, confirmation, and sequence context surrounding the lesion site (Buterin *et al.* 2000, Cai *et al.* 2007, 2009, Kropachev *et al.* 2009, Cai *et al.* 2010a). Various studies highlight how sequence context can influence NER efficacy by impacting DNA structure and subsequent recognition processes (Cai *et al.* 2009, 2010a, 2010b, 2012). Paul *et al.*, reveal that distinct resistances to NER-induced untwisting/bending renders certain contexts more likely to access conformations required for successful repair, thereby proposing a sequence-dependent propensity and trajectory for DNA ‘unwinding’ (Paul *et al.* 2021). Furthermore, we previously reported sequence-dependent variability in DNA distortion at BPDE adduct sites linked to lung cancer in *TP53* (Menzies *et al.* 2015). Therefore, the observed disparity in adduct site repairability may arise from sequence-driven modifications in DNA structure. However, the true extent to which sequence context influences distortional profiles induced by BPDE, and consequently, NER efficiency, remains a subject of debate, prompting the need for continued study.

**Figure 2.**
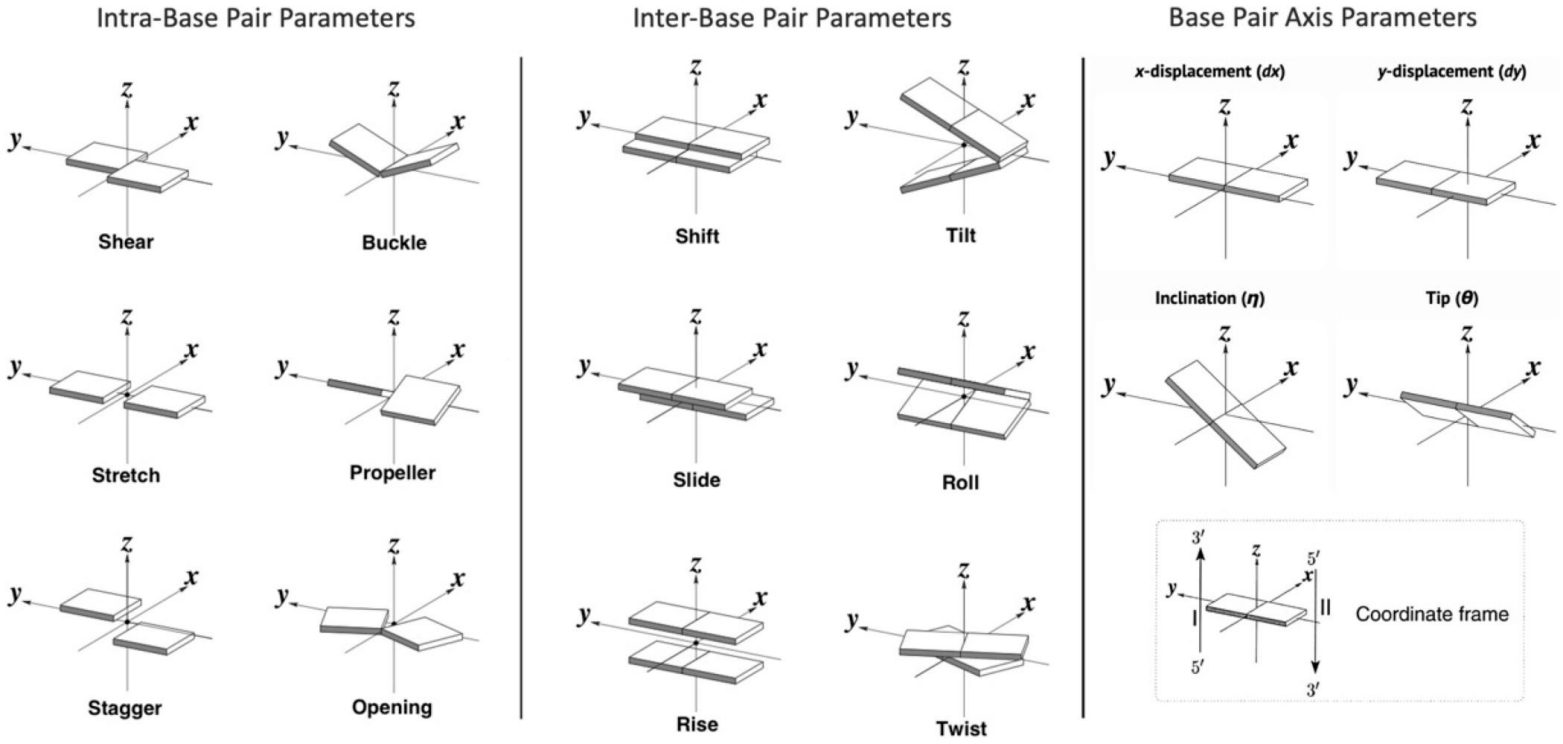
Schematic representation of base-pair parameters. Intra-base pair parameters describe the angular or distance relationships between nucleotides on opposite strands, with Shear, Stretch, and Stagger being translational parameters, and Buckle, Propeller, and Opening being rotational parameters. Inter-base pair parameters describe the angular or distance relationships between successive nucleotide base pair steps. Shift, Slide, and Rise are categorized as translational, while Tilt, Roll, and Twist are categorized as rotational. Axis base parameters indicate the position or orientation of the base pair relative to the helical axis, reprinted with permission from Neculeic Acids Research Lu and Olson (2008).

Furthermore, analysing DNA base pair parameters is essential for detecting subtle alterations in helical structure caused by DNA adducts. These metrics hold particular significance in our study due to the nature of the adducts under investigation, which form covalent bonds with the DNA duplex, resulting in nuanced yet consequential changes at the atomic level. These alterations have the potential to influence the capacity of DNA adducts to instigate mutations through comprising NER recognition processes, as proposed by computational models such as that of Cai *et al.* (2012). The realm of base pair parameters encompasses intra-base pair interactions, delineating the relationships within individual base pairs, as well as inter-base pair step parameters, which characterize the arrangement of successive base pairs within a step. These parameters are further subdivided into rotational and translational categories, as visually depicted in Fig. 2, respectively, with exhaustive descriptions provided in Supplementary Table S4.

We sought to integrate the realms of molecular dynamics (MD) and machine learning (ML) to formulate a model capable of defining the precise features that promote the differing repair efficiency of BPDE adducts observed in *TP53* during lung tumorigenesis. We hypothesized that by the inclusion of ML methodologies we would significantly amplify the range of hotspots sites under analysis, enabling the comparison of mutation spectra within single or multiple gene contexts. Our initial aim was to determine whether helical information acquired from MD simulation could indeed be used to isolate sequence dependant features that aberrate the NER of BPDE lesions in *TP53*. Upon achieving this, our subsequent goal was to ascertain the model's efficacy in utilizing this information in predicting the mutation status of unseen helical data from alternative gene contexts that are recognized to undergo BPDE-induced mutations including *cII* and *lacZ*. The knowledge gained could help provide more information surrounding the aetiology of the disease and allow for the development innovative anti-cancer therapies aimed at reinstating healthy NER function at these sites.

## 2 Methods

### 2.1 DNA sequences

*TP53*, *cII*, and *lacZ* were selected as suitable models to explore the influence of sequence context on codon mutability. For each gene context, MD simulations were performed on 25-mer duplex sequences encompassing either mutation hotspot or nonhotspot sites in the presence of the 10S (+)-trans-N2-BPDE-dG adduct (Supplementary Tables S1–S4). Each sequence consisted of a methylated cytosine at position six, and guanine at position 7, the ‘meth’ dataset was also methylated at all CpG sites. For all sequences, position 7 defines a known location of BPDE binding, and subsequently, the mutation site for any hotspot sequence. Simulations were performed on both adducted and nonadducted duplexes. Sequences were defined as having a mutation hotspot at position 7 if the frequency of G:C > T:A substitutions observed at this site was significantly elevated compared to the expected frequency. *TP53* codon 282 is characterized by a G:C > A:T substitution, however, given its extremely high incidence in lung tumours, was included in our analysis. Mutational data for *TP53*, *cII*, and *lacZ* gene contexts were obtained from Forbes *et al.* (2015), Yoon *et al.* (2001), and Beal *et al.* (2015), respectively.

### 2.2 Starting structures

All sequences, and methylation sites, were assembled or edited using the Builder toolbox in PyMOL (Schrödinger and DeLano 2020). A nuclear magnetic resonance (NMR) structure for the 7S,8R,dihydroxy-9R,10S-epoxy-7S,8R,9R,10S (+)-trans-anti-B[*a*]PDE DNA adduct (PDB ID: 1AXO) was utilized to assemble carcinogen-bound duplexes (Feng *et al.* 1997). This PDB file contains coordinates for six structures obtained from a relaxation matrix refinement, and the first structure was chosen.

### 2.3 Molecular dynamics simulations

All simulations were run in triplicate using the GROMACS package and Amber99 force field with backbone torsion modifications, bsc1 (Abraham *et al.* 2015, Case *et al.* 2023). Forcefield parameters for BPDE-bound to guanine are detailed in (Menzies *et al.* 2015). Partial charges for the methylated cytosine bases were calculated using Atomic Charge Calculator and subsequently added to the forcefield (Raček *et al.* 2020). All structures were placed in a cubic box, solvated using the explicit water model TIP3P, and neutralized with the appropriate number of ${\text{Na}}_{\;}^{+}$ions. Temperature coupling thermostat was applied using v-rescale, and Particle-mesh Ewald (PME) was used for long-range electrostatics. Simulations were carried out using the NPT ensemble, with periodic boundary conditions, at a temperature of 310.15°K, and a pressure of 1 atm. All simulations were performed using three-stage process: steepest descent energy minimization with a tolerance of 1000 kJ mol^−1 ^nm^−1^, followed by a two-stage equilibration process, each one 50 000 steps in length with a time step integration of 0.002 ps, making a total of 100 ps; and an MD stage run for a total of 100 ns.

### 2.4 DNA structural parameters

To evaluate the resulting distortion, we analysed seventeen distinct DNA structural parameters for each base pair, derived from individual MD trajectories. These parameters encompassed both intra-base pair characteristics—shear, propeller, buckle, stretch, stagger, and opening—as well as inter-base pair attributes—twist, roll, tilt, rise, shift, and slide. Supplementing these, two translational base pair axis parameters (X and Y displacement), two rotational parameters (inclination and tip), and an axis-bend parameter to define base pair geometry relative to the helical axis. Notably, helical information for base pairs 3–23 exclusively served as the machine learning input data. Parameters were measured using Curves+ and Canal software (Olson *et al.* 2001).

### 2.5 Classifier comparison

To select an optimal classifier, we compared the predictive capabilities of 15 distinct classification algorithms. We compared the performance of Nearest Neighbours, Linear Support Vector Machine (SVM), Polynomial SVM, Radial Basis Function (RBF) SVM, Gaussian Process, Gradient Boosting, Decision Tree, Extra Trees, Random Forest (RF), Neural Network, AdaBoost, naïve Bayes, Quadratic Discriminant Analysis (QDA), Stochastic Gradient Descent (SGD), and Linear Discriminant Analysis (LDA).

To ensure a robust assessment of model performance and generalization capabilities, a 10-fold cross-validation strategy was implemented during the training phase. For each fold, the classifiers were initialized with default parameters and trained on 80% of the *TP53* dataset, with the remaining 20% reserved for testing. Each model was initialized using 10 different random seeds, generating distinct training and testing splits. Performance metrics, including accuracy, precision, recall, f1 score, train-test gap, and Area Under the Curve of the Receiver Operating Characteristic (AUC ROC), were used for a thorough evaluation. In the analysis of the ROC, classification thresholds were systematically explored for each classifier, leveraging Scikit-learn's ‘roc_curve’ function. The function defaulted to use unique score values as thresholds. The exact increments and range of threshold variations were empirically determined during the analysis, ensuring flexibility in exploring decision boundaries based on classifier behaviour.

### 2.6 Random forest and feature selection

**Figure 3.**
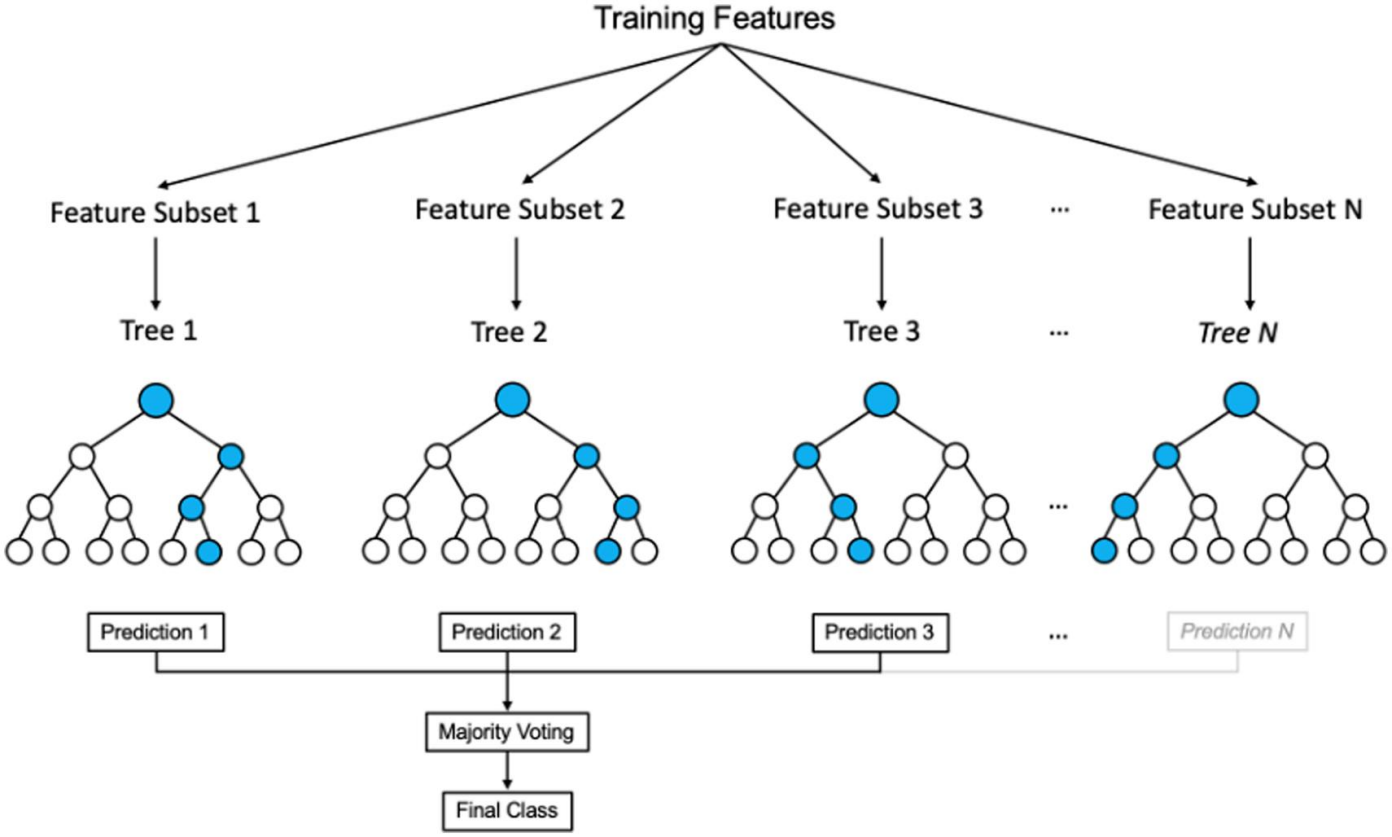
Generalized framework of a random forest algorithm.

Each decision tree in the RF was trained to predict the repair phenotype and carcinogen status of the duplexes described above, based on their structural dynamics (Fig. 3). For model evaluation, 80% of the *TP53* dataset was randomly selected as training data, with the remaining data being separated for model testing. Utilizing identical training events, the classifier was then deployed to various mutational models including *cII*, *lacZ*, and novel *TP53* datasets, where 95% of the unseen data was used for testing, ensuring a comprehensive representation of RF applicability. Optimal hyperparameters were selected using 10-fold Grid Search Optimization (GSO). Each RF model was implemented using the RandomForestClassifier module from the Python machine learning library scikit-learn (Fabian Pedregosa 2011). RF models were run using the same 10 random seeds to ensure reproducibility across each validated model. RF models were also run using initialization with 10 different random seeds.

Feature selection sought to quantitatively identify and isolate a subset of features deemed crucial for the classification process. To do so, we used three distinct feature selection techniques to ensure a comprehensive and insightful exploration of feature importance. Utilising RF feature importance with 10-fold cross-validation, recursive feature elimination with 10-fold cross-validation, and lasso (L1) regularization, we discerned the most informative features for our analysis. From each technique, the top 10% (36) features were extracted, each feature was assigned a score ranging from 36 (indicating the highest importance) to 0 (reflecting the lowest importance), features receiving a score of 0 were excluded. The scores obtained from each technique were then consolidated to create a comprehensive ranking of features, ranging from most important to least.

## 3 Results

### 3.1 Data retrieval and definition of spectral investigation

Our analysis was focused on mutational spectra of three distinct gene contexts—*TP53*, *cII*, and *lacZ*. Within each gene context, a significant propensity for BPDE binding unfolds across a selection of distinct DNA bases, as elucidated in Forbes *et al.* (2015), Yoon *et al.* (2001), and Beal *et al.* (2015). This coexists with site-specific variation in repair efficiency, highlighting nuanced distinctions that contribute to differences in mutational probability. We separated known BPDE binding sites into two discernible datasets in which those exhibiting elevated levels of mutational frequency constitute our positive, hotspot dataset, and sites demonstrating low mutational frequency in response to BPDE adduct formation formed our negative, nonhotspot dataset.

### 3.2 Conformational stability

**Figure 4.**
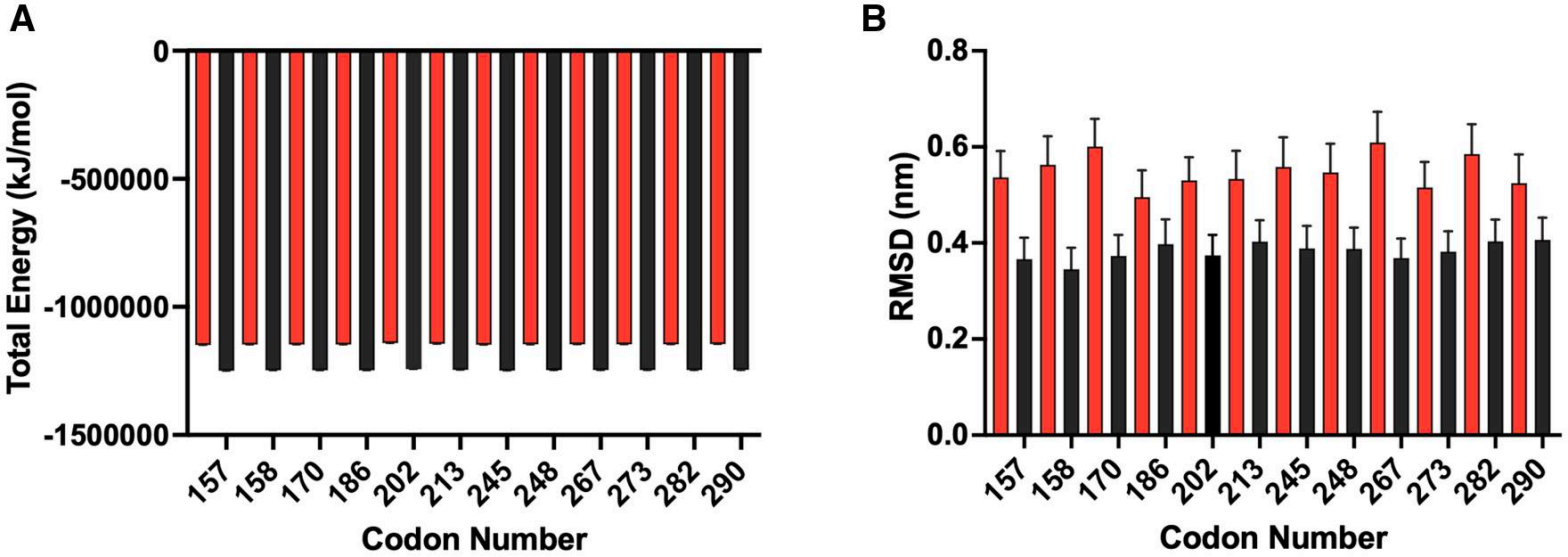
Conformational stability and flexibility of adducted and control *TP53* sequences. Total energy (A) in KJ/mol and RMSD values (B) in nm for control sequences (right hand bars) and adducted sequences (left hand bars); error bars show ± standard deviation, differences between the control and adducted sequences were significant (*P* < 0.001). Corresponding values for Val, Meth, cII, and lacZ datasets are depicted in Supplementary Figs S1–S4.

Root mean square deviation (RMSD), root mean square fluctuation (RMSF) and total energy analysis provided confidence about the stability of the simulations. Elevated RMSD values typically suggest that flexibility and movement during a simulation are likely characterized by instability. For *TP53*, total energy calculations were stable (Fig. 4A). Average RMSD values for each sequence, relative to the initial structure, provided valuable insights into the variations in flexibility observed throughout the 100 ns simulation period. As depicted in Fig. 4B, unbound *TP53* duplexes showed a mean RMSD of 3.83 Å ± 0.81, while adducted sequences exhibited a higher mean RMSD of 5.50 Å ± 1.10. BPDE adducts are known to disrupt the helical structure, resulting in increased fluctuation and mobility. Similarly, root mean square fluctuation (RMSF), which provides a measure of residue-level displacement relative to the reference structure, report a partial increase in conformational flexibility among lesion bound (0.30 Å ± 0.08) relative to unbound duplexes (0.27 Å ± 0.07) (Supplementary Fig. S5A). Such analyses also reveal localized dynamics surrounding the lesion of adduct bound duplexes. For all simulations, base 6 was shown to be conformationally diverse (0.41 Å ± 0.11), whilst its corresponding base (base 45), was shown to demonstrate minimal flexibility (0.21 Å ± 0.02). These observations were anticipated given that BPDE’s orientation permits the disruption of native interactions within the immediate vicinity of the lesion site, particularly those in the 5’ direction. Despite this, whole structure RMSD and RMSF showed little variation during the simulation period of 10–100 ns, which prompted the exclusion of the initial 10 ns from all subsequent analyses.

### 3.3 Classifier comparison

Helical parameters were extracted using Curves+ and Canal, these offer an extensive array of helical and backbone parameters, including a curvilinear axis and parameters detailing the positioning of the bases relative to this axis (Olson *et al.* 2001). Only data generated within the period of 10–100 ns was evaluated to accommodate fluctuations associated with system equilibration. A total of 17 distinctive parameters for every DNA base pair were extracted. To address heightened instability stemming from the absence of stacking interactions, data for two bases at each terminus was excluded from our analysis. As such, each sample trajectory yielded 357 unique features per duplex. Given that the 12 *TP53* sites under investigations were simulated in triplicate, and in the absence or presence of the adduct, our initial dataset was comprised of 25 704 unique observations derived from 72 simulations.

Rather than averaging the features across the three replicates, we preserved the raw data to capture the full extent of variation within the system. This approach allowed us to observe and analyse the intrinsic variability and subtle differences between individual simulations. Furthermore, we enhanced our ability to identify patterns and outliers, offering a more nuanced understanding of the helical parameters by maintaining the raw data. This comprehensive dataset provided a robust foundation for our four-class classification problem, enabling a detailed exploration of the structural dynamics under variable sequence contexts.

To select an appropriate classifier, we first compared the prediction results of 15 unique classification algorithms on the *TP53* dataset. Each classifier underwent training with 80% of the helical data, with the remaining 20% allocated for testing. Notably, default hyperparameters, as curated by Scikit-Learn developers, were applied during the training of each model. Model performance evaluation encompassed accuracy, precision, recall, f1, area under the curve (AUC), and receiver operating characteristic (ROC) scores and included a 10-fold cross-validation strategy. Default hyperparameters served as a practical starting point for model comparison but also streamlined the evaluation of diverse models, aligning with the common practice of utilizing default settings for preliminary assessments before venturing into hyperparameter fine-tuning.

**Figure 5.**
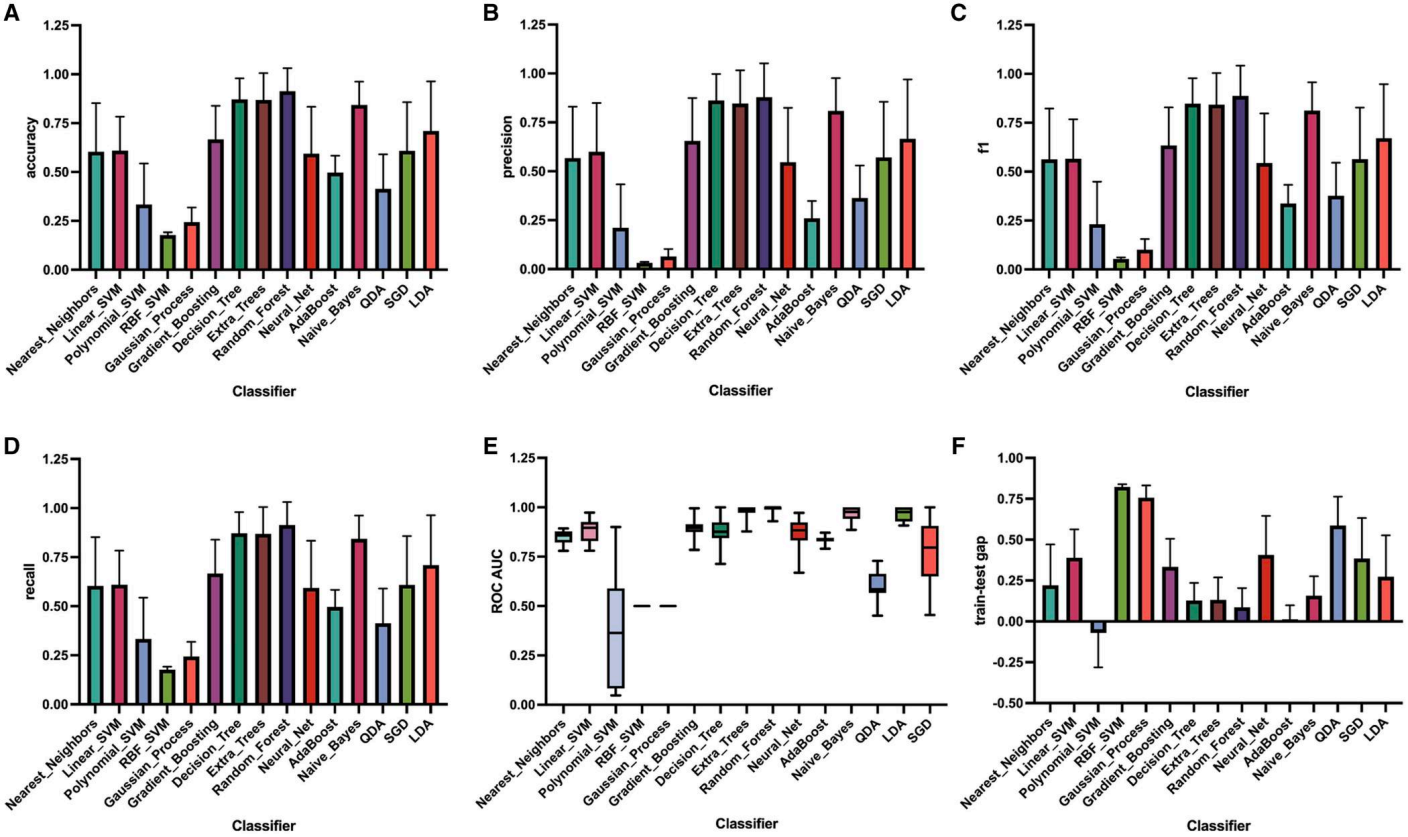
Evaluation results of 15 classification algorithms using accuracy (A), precision (B), f1 (C), recall (D), ROC AUC (E), and training-test gap scores (F). Error bars represent ± standard deviation for panels (A) to (D) and indicate the range of ROC AUC scores in panel (E), with the median score depicted by a solid black line. All evaluations were conducted using a 10-fold cross-validation strategy. In addition, each model was initialized using varied random seeds.

Among the 15 algorithms, decision tree-based models (Decision Tree, Extra Trees, and RF) were by far the most prominent, demonstrating advanced results ranging from 0.84 to 0.91 across each evaluation metric (Fig. 5A–D). Kernel machines (Polynomial SVM, RBF SVM, and Gaussian Process), conversely, encountered substantial challenges during the classification process, resulting in a notable decline in accuracy, precision, recall, f1, and, in the case of Polynomial SVM, considerable variation in ROC AUC (Fig. 5E). As shown in Fig. 4A–F, the RF algorithm exhibited the best discriminatory power, with all mean evaluation scores within the range of 0.88–0.99. ROC AUC values displayed minimal range (±0.035), highlighting the models consistent and robust discrimination across different scenarios (Fig. 5E). We observed that, in comparison to all other well-performing algorithms, the RF model exhibits the smallest training-test gap (Fig. 5F). This finding confirms the superior generalization capacity of the RF model, solidifying its standing as the optimal classifier for our study.

### 3.4 Grid search optimization

To identify the optimal hyperparameter configuration of our model, we used Grid Search Optimization (GSO). Crucial parameters, including bootstrap, min_sample_leaf, max_features, and n_estimators, significantly influence the performance of the RF classifier. Utilizing GSO, we transformed these parameters into a grid with a defined spatial range and explored all points to obtain the global optimal solution. The architectural specifications of the optimized RF model encompassed setting the number of decision trees to 89 and constraining the depth of each tree to 4 levels. We used the square root of the number of features at each split, and defined criteria for leaf nodes, specifying minimum samples per leaf (1), and the minimum samples required to split an internal node (2). Furthermore, for this configuration, bootstrap was disabled, ensuring the creation of a diverse set of trees.

**Figure 6.**
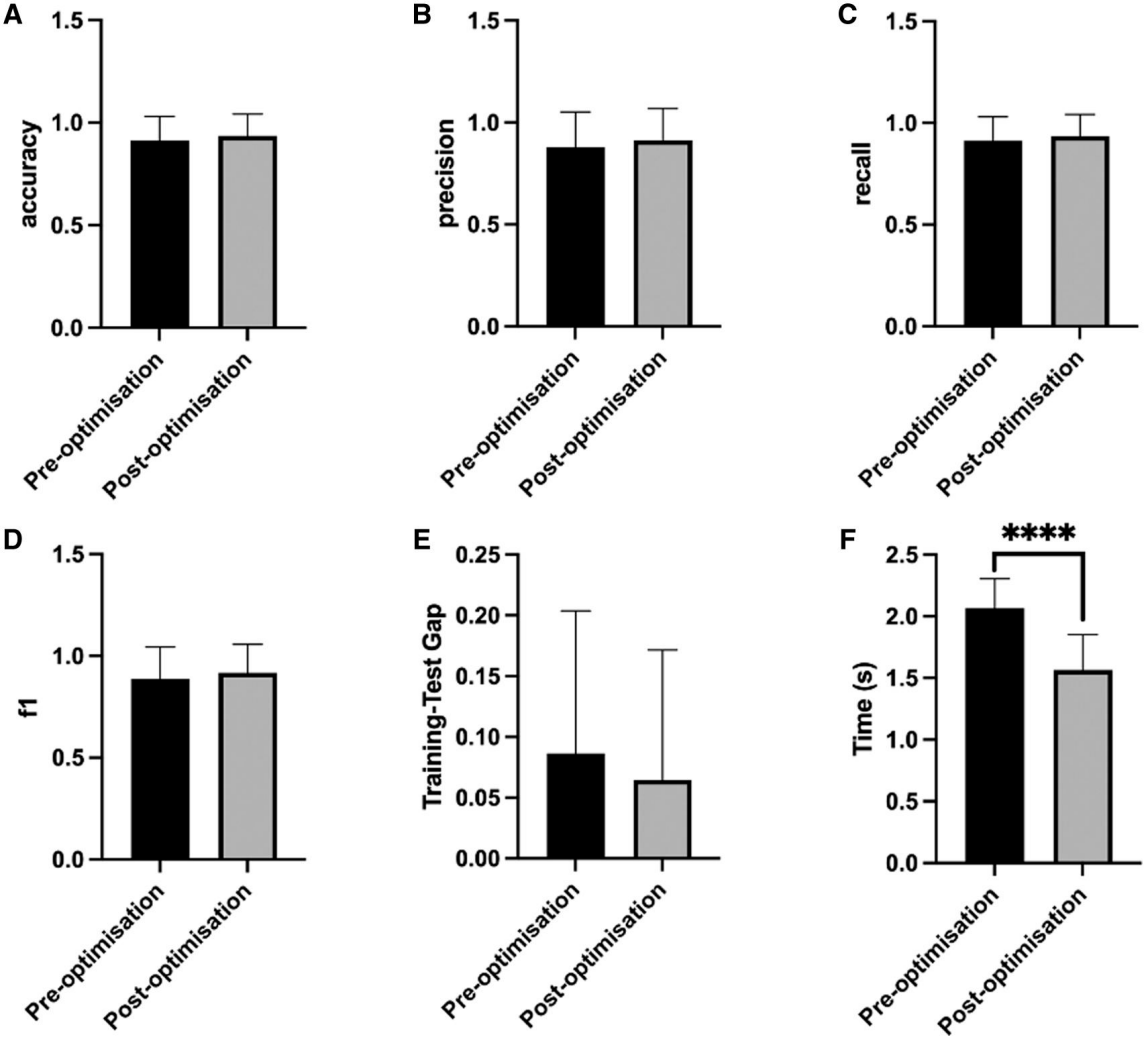
RF optimization by GSO. Bar chart illustrating the averaged accuracy (A), precision (B), recall (C), f1 (D), train-test gap (E), and execution time (F) scores before and after optimization. Pre-optimization scores are shown in black, while post-optimization scores are depicted in grey. Error bars indicate ± standard deviation.

Incorporating novel GSO parameters into the existing RF algorithm was shown to enhance model performance, yielding a 3.06% increase in model accuracy and 12.63% improvement in precision (Fig. 6A and B). Hyperparameter tuning also induced modest improvements in recall, f1, and training-test gap scores (Fig. 6C–E). This not only bolstered overall accuracy but also honed the model’s ability to detect positive cases while mitigating instances of false negatives and false positives. Further to this, GSO was shown to significantly improve the model’s operating speed (*P* < 0.0001) (Fig. 6F). This is to be expected given that GSO typically accelerates convergence during model training.

### 3.5 *TP53* context

To bolster the robustness and refine the predictive capabilities of our model, we opted to leverage the knowledge acquired during the original training phase to classify two previously unseen datasets: a designated validation dataset, termed ‘Val’, sourced from the re-simulation of *TP53* starting structures used previously, and a secondary dataset representing *TP53* sequences characterized by full CpG methylation, termed ‘Meth’. This multifaceted strategy provided insights into the generalization capacity of the model by exposing it to a more extensive classification problem, offering a comprehensive view of the conserved dynamics within each class in the context of *TP53*. RMSD and RMSF profiles consistently depict stable duplexes across the 90 ns analysis period. Despite a minor increase in both metrics in the instance of homomethylated control duplexes, the regions of highest flexibility were restricted to base 6 (Supplementary Fig. S5B and C). Once more, the model underwent a meticulous evaluation, using essential metrics including accuracy, precision, f1 score, and recall. The outcomes of these evaluations, representing the model's performance across 10 distinct training and test splits initialized by different random seeds, are presented in Table 1. Feature scoring was then applied exclusively to adduct-bound sequences to deduce the predictive utility of each helical parameter for sample classification.

**Table 1.** Mean accuracy, precision, f1 score, and recall across 10 distinct training and test splits.

Context	Accuracy	Precision	f1	recall
*TP53*	0.935	0.913	0.917	0.935
Val	0.986	0.987	0.986	0.986
Meth	0.928	0.931	0.926	0.876

Table 1 reveals a prominent improvement in our model’s performance following its deployment on the validation dataset. The model achieves a near perfect performance, showcasing an average recall, accuracy, and f1 score of 0.986, coupled with an average precision score of 0.987. Notably, precision and f1 metrics exhibit an approximate 7% increase, indicating improved accuracy in positive predictions and a refined equilibrium between precision and recall. Despite added complexity in the form of CpG methylation, our RF model maintains exceptional classification performance, achieving high scores in precision (0.931), f1 (0.926), and accuracy (0.928). Prediction recall scores, however, experience a marginal decline, registering a modest value of 0.876, indicating potential challenges in accurately identifying positive instances in the event of differentially methylated training and test datasets.

To pinpoint the precise parameters governing the classification of helical data we harnessed three distinct feature selection methodologies: Recursive feature elimination with cross-validation (RFECV), RF feature importance, and Lasso (L1) regularization. Identified features were then ranked through a unified scoring function, before being combined to present a holistic understanding of the pivotal features contributing to the classification process.

Feature scoring unveiled notable alignments in parameter types defined as pivotal during the classification process. Parameters illustrating the rotation of a base pairs/base pair axes took precedence among the top 10% of scored features, contributing >61% of the highlighted components (Table 2). A bias was unveiled towards the axis-displacement parameter tip, constituting 25% and 19% of the identified features in the *TP53* and Val datasets, respectively. Inclination and tip angles mirror the orientation of base-pair planes concerning the helical axis. This observation naturally prompts the anticipation that parameters governing base step planarity such as roll, tilt, and buckle angles would also exhibit elevated scoring. Our dataset validates this expectation by showcasing a substantial prevalence of these parameters within the upper echelons of the top 10% of featured attributes.

**Table 2.** Top 10 helical parameters within *TP53*-related datasets identified by feature scoring.^a^

Rank	*TP53*	Val	Meth
1	14—propeller	23—rise	14—propeller
	±4.91	±0.08 Å	±5.40°
2	23—rise	14—propeller	21—axis bend
	±0.05 Å	±4.72°	±3.26 Å
3	10—axis bend	23—tip	13—propeller
	±1.64 Å	±118.12°	±15.15°
4	10—tip	13—propeller	14—opening
	±118.80°	±3.37°	±3.01°
5	11—shift	11—shift	11—tip
	0.18 Å	±0.21 Å	±107.88°
6	10—X displacement	16—axis bend	23—tip
	±0.03 Å	±2.38 Å	±108.94°
7	14—stretch	10—tip	6—propeller
	±0.45 Å	±118.05°	±99.14°
8	13—stagger	23—shear	12—slide
	±0.03 Å	±1.56 Å	±0.44°
9	17—axis bend	11—propeller	13—axis bend
	±2.66 Å	±5.50°	±10.50 Å
10	23—tip	10—X displacement	13—slide
	±108.00°	±0.03 Å	±0.58°

a Abbreviations explained in Supplementary Table S4.

These trends extended to the methylation dataset, with tip, buckle, axis-bend, propeller, and opening continuing to dominate as primary helical attributes. Notably this set of five parameters accounted for an impressive 72% of identified features, with tip emerging as the most influential contributor, commanding 19% of the overall proportion. Bases 10, 14, and 23 maintained their position as the highest-scoring base positions across all three *TP53*-based datasets. However, due to their distal location from the lesion site, it remains challenging to draw definitive conclusions regarding the precise significance of these positions in relation to the observed distortion.

### 3.6 Alternative gene context

After deploying our RF model and feature scoring pipeline, we used the approach to deduce the relative discriminatory potential of our training data concerning hotspot and nonhotspot samples in alternative gene contexts and identify potentially conserved helical parameters likely to contribute to the repair phenotype. As outlined in Supplementary Tables S2 and S3, our analysis identified seven well-established BPDE binding sites within the *lacZ* and *cII* genes. Five sites in each gene displayed elevated mutational frequencies, designating them as hotspot sites, while the remaining two were classified as nonhotspot sites due to their lower mutational frequency. Duplex stability was again evaluated using RMSD and RMSF analysis with duplexes not containing BPDE demonstrating the greatest conformational stability (Supplementary Fig. S5D and E). No significant variation, however, in the RMSF profiles of bound duplexes was observed relative to those of *TP53* and Val. Subsequently, we harnessed the insights gained from 80% of the *TP53* dataset to guide the investigation of two supplementary unseen gene contexts with established BPDE-binding sites, the results of which are depicted in Table 3.

**Table 3.** Mean accuracy, precision, f1 score, and recall across 10 distinct training and test splits.

Context	Accuracy	Precision	f1	Recall
*cII*	*0.628*	*0.737*	*0.639*	*0.628*
*lacZ*	*0.788*	*0.884*	*0.800*	*0.788*

As anticipated, deploying the RF model on structural data acquired from duplexes with unseen base compositions yielded a noticeable decline in classifier performance. Specifically, in the context of *cII*, accuracy, f1, and recall demonstrated substantial reductions of approximately 35%–36% relative to that observed upon Val deployment. Remarkably, despite these changes, precision scores remained steadfast at 0.737, highlighting the model’s classification potential amid shifting sequence contexts. Similarly, the RF model’s predictive capacity encountered limitations when applied to the *lacZ* dataset. The model accurately predicted a significant proportion of samples (78.8%), achieving a notable precision rate of 88.4%. It’s worth noting that recall (78.8%) slightly lagged precision, hinting at potential cases where positive instances might not have been precisely identified. These results align with our expectations, underscoring the model's susceptibility to overtraining and heightened sensitivity to sequence intricacies arising from training the model on a single-gene context.

Examining the top-ranked features in *cII* and *lacZ* datasets reaffirmed an emphasis on rotation-specific parameters. Parallel to that observed in *TP53*, the tip parameter remained dominant, constituting 22% and 28% of identified features in *cII* and *lacZ* datasets, respectively (Table 4). Similarly, an abundance of parameters associated with base step rotation, including buckle, incline, propeller, and opening, persisted, accounting for 58% (*cII*) and 69% (*lacZ*) of the top 36 features in each dataset. Despite minor alterations in the distribution of rotational parameters, these findings closely resembled those within *TP53*-specific contexts, accentuating the emphasis on rotational parameters as primary contributors to the discrimination process. It’s worth noting that the positioning of these parameters exhibited notable deviation from *TP53* results, aligning with our expectations due to the change in base contexts. Collectively, these insights underscore the fundamental role of rotational parameters in shaping the model’s discriminative prowess across diverse gene contexts.

**Table 4.** Top 10 helical parameters within non-*TP53* datasets identified by feature scoring.^a^

Rank	*cII*	*lacZ*
1	17—roll	10—incline
	±2.38°	±21.00°
2	15—incline	9—buckle
	±16.09°	±4.2°
3	13—tip	17—tip
	±112.53°	±106.6°
4	16—axis bend	20—stretch
	±3.02 Å	±5.24 Å
5	8—slide	16—tip
	±0.26 Å	±131.70°
6	15—axis bend	22—tilt
	±2.29 Å	±3.20°
7	15—tip	10—buckle
	±96.20°	±2.90°
8	11—Y displacement	17—buckle
	±0.10 Å	±1.80°
9	21—twist	17—stagger
	±7.99°	±0.30°
10	8—incline	5—tip
	±61.25°	±84.09°

a Abbreviations explained in Supplementary Table S4.

## 4 Discussion

The intricacies of DNA structure and its dynamic flexibility constitute essential features of sustained eukaryotic life (Abeel *et al.* 2008, Rohs *et al.* 2009, Yella *et al.* 2018, Yella *et al.* 2020). Paradoxically, the attributes that underscore DNA’s functionality can also support detrimental processes, such as those driving tumorigenesis (Feng *et al.* 2002, DiGiovanna and Kraemer 2012, Menzies *et al.* 2015). Prior research has exposed the influence of BPDE on damaged base conformation and DNA bending however thorough investigation into the structural factors driving mutations across multiple gene contexts has not been pursued (Donny-Clark and Broyde 2009, Menzies *et al.* 2015, Menzies *et al.* 2021). We conducted a focused investigation to uncover how sequence context influences the structural modifications induced by BPDE adducts at mutation sites within *TP53*, *cII*, and *lacZ* genes. From this we identified conserved patterns in helical distortion among regions of elevated mutational frequency, offering novel insights that can aid in the development of anti-cancer therapies.

We faced a fundamental challenge posed by the high dimensionality of our dataset, with the number of features surpassing that of the available samples, a circumstance commonly known as the ‘curse of dimensionality’. This phenomenon introduces notable challenges, including heightened model complexity and an increased susceptibility to overfitting. We exercised caution and strategic deliberation to extract meaningful insights from our data. Acknowledging these challenges we opted to use a supervised machine learning (ML) approach. With various ML methodologies offering unique advantages for analysing such datasets, we embarked on an evaluation of 15 distinct ML classifiers, aiming to pinpoint the most appropriate model for our specific framework. As anticipated, decision tree-based models, including Decision Tree, Extra Trees, and RF, consistently outperformed other models across different class variables. Although the performance differences among these three models was marginal, it is noteworthy that the RF algorithm consistently achieved the highest scores for all evaluation metrics, and so became our method of choice for further analysis.

We leveraged a well-established feature in the field of cancer biology—the *TP53* mutation spectrum—to evaluate our model’s proficiency in classifying previously unseen helical data (Forbes *et al.* 2015). As anticipated, model deployment on the validation dataset yielded the highest prediction scores. However, given the consistency in starting structures for both *TP53* and Val, the simulations have the potential to produce the same ensemble averages if sufficiently converged, despite being equilibrated with different seeds. As such, we conducted an additional validation step to evaluate the convergence and similarity of the sampled space in the *TP53* and Val datasets. Specifically, we constructed a new *TP53* dataset by excluding 10% of the original simulation time to determine if this exclusion would impact the model's classification performance. Consistent with prior experiments, we utilized 80% of this new *TP53* dataset for model training and subsequently deployed it on 95% of the original Val dataset. The final classification remained largely unchanged, showing only a minor decline of approximately 0.2%–0.3% in each evaluation metric (Supplementary Table S5). This minimal change in performance indicates that the key information required for effective dataset distinction is retained even when a portion of the simulation data is excluded. Consequently, this analysis provides strong evidence that the simulations are sufficiently converged and that the sampled space is adequately similar across different portions of the simulation. Moreover, this finding underscores the robustness of our results, demonstrating that the classification accuracy is maintained within the constraints of our 100 ns simulations, irrespective of further sampling. However, despite the convergence mentioned previously, we observed variations in feature selection in terms of both parameter type and the base number at which the features are deemed crucial. This observation indicates that, although there appears to be convergence in simulations among the *TP53* and Val datasets, distinct motions among the two enable the classification model to differentiate them effectively. This distinction is critical as it underscores the importance of analysing the differences between runs performed on the Val dataset compared to their replicates present in the training dataset.

Despite maintaining exceptional performance on the validation data, the classifier exhibited partial regression when applied to the methylation dataset. The influence of CpG methylation on DNA flexibility and its potential repercussions on NER have been a subject of inquiry. Several studies have indicated that regions with heightened cytosine methylation enhance NER function at BPDE lesions (Derreumaux *et al.* 2001, Colgate *et al.* 2003, Muheim *et al.* 2003). Given our models adaptability to methylation data, we suggest that cytosine methylation has limited influence on the observed dynamics, and therefore, on NER efficiency. This aligns with the notion that all *TP53* CpG sites within lung tissue exhibit methylation, implying that methylation, in isolation, cannot comprehensively explain the variable DNA repair rates witnessed at BPDE binding sites (Tornaletti and Pfeifer 1995).

The model’s effectiveness declined when confronted with helical data from different gene contexts. While it is commonly agreed that imbalanced datasets adversely impact classifier performance, it is possible to mitigate population bias and improve overall model training by combining all available data (Seiffert *et al.* 2010). However, this strategy can complicate the assessment of information specific to the training dataset. Thus, we made the deliberate choice to maintain the segregation of data. It's essential to note that our input data had some limitations, particularly in the absence of information regarding nonhotspot sites. To address this, for *lacZ* we defined nonhotspot sites based on reported codon mutational frequencies upon BPDE exposure, categorizing them as sites with lower mutational frequencies compared to those of defined hotspots (Beal *et al.* 2015). Similar limitations were observed in the *cII* dataset, where information on nonhotspot sites was limited (Yoon *et al.* 2001). Given these constraints in our nonhotspot data, we refrain from speculating whether this decline in performance solely stems from sequence context or if it's compounded by the inherent dataset imbalance present in *cII* and *lacZ* datasets.

A recurrent and pivotal parameter during our analysis was tip, denoting the angular displacement of the base pair around its y-axis (Olson *et al.* 2001). Notably, this parameter exhibited remarkable prominence, emerging as the preeminent feature within each of the five helical datasets. Alternative rotational parameters, including buckle, opening, and propeller also garnered high scores through our combined feature scoring approach. This outcome aligns with our expectations, considering that the collective variation in individual base/base step parameters is likely to exert a substantial impact on overall axis displacement. Our findings resonate with prior molecular modelling studies, which highlight the effects of DNA bending at established sites of DNA damage. Using multivariate statistical analysis, Menzies *et al.*, report that buckle and opening parameters significantly contribute to the structural differentiation of adducted sequences in *TP53* (Menzies *et al.* 2015). Paul *et al.*, using crystallographic and fluorescence lifetime (FLT)-based conformational studies, show that elevated values for slide, roll, and twist parameters can aid DNA opening events during NER, highlighting a distinct interplay between rotational and transitional parameters (Paul *et al.* 2021). As such, we refrain from speculating that rotational parameters alone govern NER functionality, instead highlighting the importance of parameters, including tip, as a potent discriminator of hotspot and nonhotspot sites.

A global analysis of feature importance scores also offered insights into the specific positions of base pairs with significant predictive utility. Results highlighted that bases of intrinsic importance to the classification process were distributed across the entirety of the 23-mer region under analysis. Moreover, each specific gene context exhibited a distinct importance profile, emphasizing the context-dependent nature of these critical bases. This observation aligns with previous studies indicating that sequence context, even more distal to adjacent bases, can exert influence over local distortion and, consequently, NER capacity (Cai *et al.* 2010a, 2010b). The specific mechanism and extent to which distal sequence affects NER, however, remains a topic of extensive debate.

In the NER pathway, the crucial processes of lesion recognition and initiation are instigated by the heterodimeric XPC–RAD23B–CETN2 complex, hereafter referred to as XPC. Using thermal energy alone, XPC scans the genomic landscape for irregularities by unwinding short segments of DNA in a nonspecific manner, a process aptly referred to as the interrogation mode (Chen *et al.* 2015). Local distortion or destabilization induced by a DNA lesion increases XPC residency at the damage site, allowing for the insertion of functional components and subsequent separation of the helical backbone (Gunz *et al.* 1996, Dip *et al.* 2004, Hoogstraten *et al.* 2008). Lesion recognition is an indispensable, rate-limiting step of NER, and stalled XPC is strictly required for the recruitment of transcription factor, TFIIH (Volker *et al.* 2001, Sugasawa *et al.* 2009, Li *et al.* 2015). Therefore, it is reasonable to consider that various base contexts may impart distinct preferences for strand separation, thus potentially influencing the overall effectiveness of NER and, subsequently, mutational frequency. Paul *et al.*, in evaluating the role of sequence context on DNA ‘opening’ by Rad4/XPC, highlight that regions of elevated van der Waals stacking energy may confer resistance to DNA opening (Paul *et al.* 2021). Moreover, it is widely reported that variation in stacking stabilization among both adduct types and sequence contexts can compensate for destabilizing distortions caused by these lesions and in turn, resist NER (Mu *et al.* 2012). Interestingly, it has been found that duplex stability depends linearly on G/C content, with the free energy of G/C base steps consistently lower than those of A/T or ‘mixed’ base pair steps (with one G-C and one A-T pair) (Zacharias 2020). Given that mutations are AT-biased in eukaryotes, it's conceivable that regions with high GC content might confer resistance to NER processes due to their enhanced thermodynamic stability, primarily governed by base stacking van der Waals interactions (Petrov and Hartl 1999, Lynch *et al.* 2008, Lynch 2010).

Analysis of *TP53*, *cII*, and *lacZ* mutational signatures appear to echo these insights. By comparing a 10 bp region spanning the lesion site (base pairs 2–12), we observe significant statistical disparities in the G/C content between hotspot and nonhotspot sites in *TP53* and *lacZ* (*P* < 0.005), (Supplementary Tables S1 and S3). In contrast, no such distinction is evident within the *cII* gene (Supplementary Table S2) potentially contributing to the observed limitations in classifier performance during *cII* deployment. While it is vital to explore the stacking profiles of adduct-bound duplexes in the specific sequence contexts discussed, our results strongly indicate that, in the case of BPDE adducts, there exists a substantial preservation of features distinguishing hotspot and nonhotspot sites, primarily governed by variations in G/C content.

While our current analysis provides valuable insights into the factors influencing site-specific variations in DNA repair, it is crucial to acknowledge the limitations imposed by the availability of mutational data. The well-established carcinogenic effect of PAHs, including B[*a*]P, has been extensively documented, linking exposure to an increased risk of various cancers such as lung, skin, and bladder cancer (Bosetti *et al.* 2007, Rota *et al.* 2014, Ifegwu and Anyakora 2015, Kim *et al.* 2016). Recent studies have further suggested associations between PAH exposure and elevated risks of cancers affecting the larynx, kidney, prostate, breast, blood (leukaemia), brain, and colon. Despite the recognized implication of the 10S (+)-trans-N2-BPDE-dG DNA adduct in human diseases, information regarding its repairability at sites linked to these cancers remains limited, constraining the number available gene contexts for analysis (Korsh *et al.* 2015, Hamidi *et al.* 2016, Petit *et al.* 2019, Amadou *et al.* 2021).

In our study, the conserved nature of findings within the context of single-gene training is promising; however, addressing limitations related to data availability is essential for model refinement. The absence of comprehensive kinetic data on DNA repair rates in the *cII* and *lacZ* gene contexts poses significant challenges. While *TP53* hotspots and nonhotspot sites were designated based on mutational frequency, BPDE binding assays, and supporting kinetic data on DNA repair, the lack of such data for *cII* and *lacZ* genes introduces uncertainty in hotspot and nonhotspot site identification, potentially contributing to the reduced classification performance observed upon model deployment. In addition, both the *cII* and *lacZ* datasets consisted of seven contexts, with five being poorly repaired sites and two being well-repaired. This uneven distribution, especially when compared to an evenly balanced training dataset, introduces class imbalance, and likely causes the model to become biased towards the more frequent poorly repaired contexts. This imbalance significantly impacts the model's ability to accurately predict the less frequent well-repaired contexts, resulting in poorer performance.

Moreover, our analysis shows that rotational parameters dominate feature selection predictions, highlighting their importance in discriminating hotspot and nonhotspot sites in both *cII* and *lacZ*. However, the bases from which these parameters are extracted vary relative to *TP53*, indicating that changes in base context influence this variation. It would be interesting to investigate whether obtaining a more balanced test set would modify this feature selection process, providing a more comprehensive elucidation of the major and minor fluctuations of hotspot and nonhotspot sites in alternative gene contexts. To enhance the model's predictive capacity, future research should focus on obtaining comprehensive kinetic data across a broader spectrum of gene contexts, allowing for a more accurate understanding of DNA repair dynamics and mitigating overfitting challenges observed with the *lacZ* and *cII* gene contexts. Importantly, however, our model's adeptness in distinguishing hotspot and nonhotspot sites in both adduct-bound and unbound DNA underscores the potential of characterizing repair efficiency solely based on DNA sequences. This suggests that inherent dynamics within these sequences likely plays a significant role in the observed poor repair.

It is also evident that a substantial number of hotspot mutations under investigation are likely influenced by selective pressures. Notably, mutations observed at *TP53* hotspot codons 245, 248, 273, and 282 are well-documented for their propensity to modify contacts, and as such, the topology of the encoded *p53’s* DNA binding domain (Baugh *et al.* 2018). This modification hinders p53's DNA-binding capabilities, leading to a compromised regulation of cell cycle progression, providing a distinct advantage in terms of survival and viability for the mutated cells. While the present model is not designed to capture these pressures, the integration of evolutionary features, such as sequence conservation and functional annotations, holds promise for refining our framework to better comprehend the forces shaping hotspot mutations. Once more, such progress awaits the expansion of mutational information across various genes exposed to BPDE to fully unlock the analytical potential of this framework.

## 5 Conclusions

Our investigation establishes the efficacy of using a ML-based approach to not only classify, but also predict biological features from a selectively curated set of helical data. This underscores the practical utility of such methodologies in the analysis of results derived from MD simulations. Within the constraints of the models developed, our findings reveal the significant role of regional helical topology in distinguishing mutation hotspot from nonhotspot sites. Particularly, we showcase that the RF classification of BPDE-bound duplexes is primarily steered by variations in base pair tip, likely stemming from differing thermodynamic stability linked to regional GC content. It's worth noting that while our analysis has provided insights into the distinctive features influencing mutational patterns in well-studied genes, a broader dataset encompassing a larger variety of sequences is essential to comprehensively understand the precise origins of these mutations. We highlight that this approach is not limited to the genes discussed above and could be applied to any gene subset of interest, including those associated with alternative human diseases. Furthermore, our methodology holds potential for screening mutation hotspot sites to assess the impact of both novel and existing drug compounds. This extends the applicability of our approach beyond fundamental research, making it a valuable tool in drug development and toxicology studies.

## Supplementary Material

vbae125_Supplementary_Data

## Data Availability

Code for comparing machine learning classifiers and evaluating their performance is available at https://github.com/jdavies24/ML-Classifier-Comparison, and code for analysing DNA structure with Curves+ and Canal using Random Forest is available at https://github.com/jdavies24/ML-classification-of-DNA-trajectories. Data is available at: https://zenodo.org/record/8310820.
