# Correction to: Utilizing biological experimental data and molecular dynamics for the classification of mutational hotspots through machine learning

**DOI:** 10.1093/bioadv/vbae140

**Published:** 2024-10-04

This is a correction to: James G. Davies, Georgina E. Menzies, Utilizing biological experimental data and molecular dynamics for the classification of mutational hotspots through machine learning, *Bioinformatics Advances*, Volume 4, Issue 1, 2024, vbae125, https://doi.org/10.1093/bioadv/vbae125

In the version originally published, the source of figure 2 was incorrectly attributed. The attribution has been corrected in the figure legend and reference list.

