# Supplementary material for: Utilizing biological experimental data and molecular dynamics for the classification of mutational hotspots through machine learning
Source: Bioinform Adv. 2024 Aug 26;4(1):vbae125. doi: 10.1093/bioadv/vbae125 (PMC11377099; doi:10.1093/bioadv/vbae125)
Supplement: vbae125_Supplementary_Data [file vbae125_supplementary_data.docx]

*
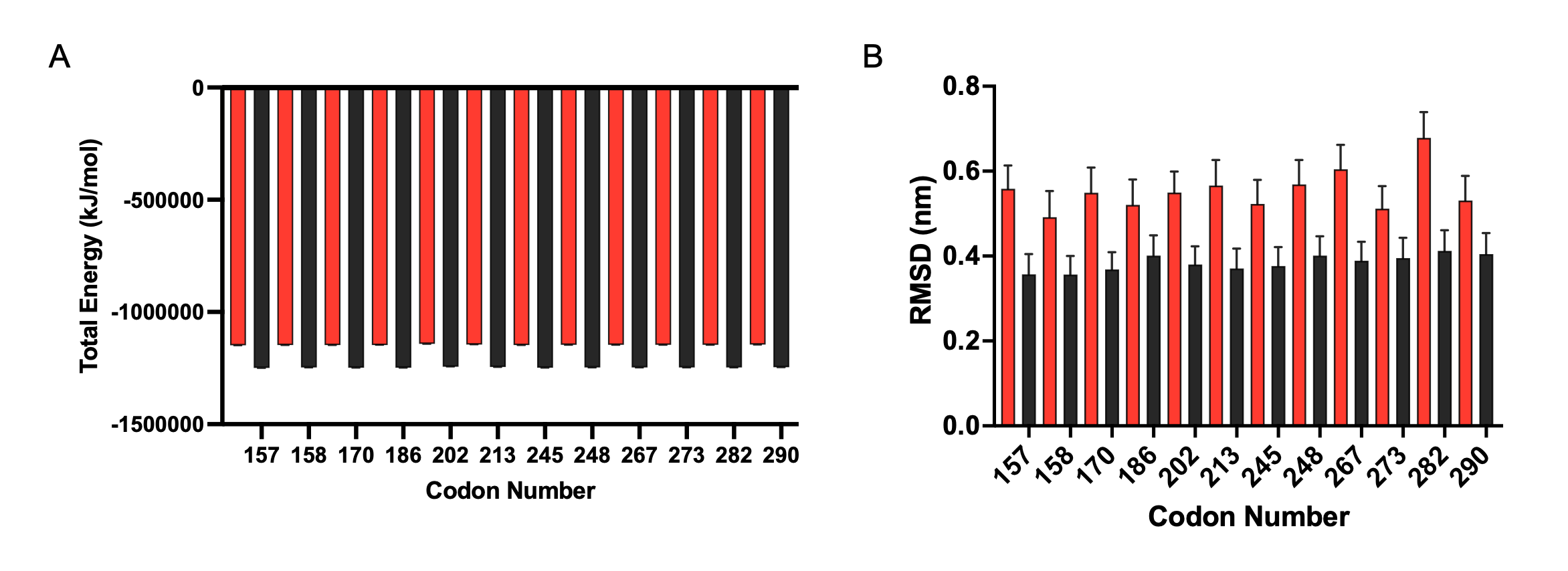
*

*Supplementary Figure S1: Conformational stability and flexibility of adducted and control Val duplexes. Total energy (****A****) in KJ/mol and RMSD values (****B****) in nm for control sequences (black) and adducted sequences (red); error bars show ± standard deviation.*

*
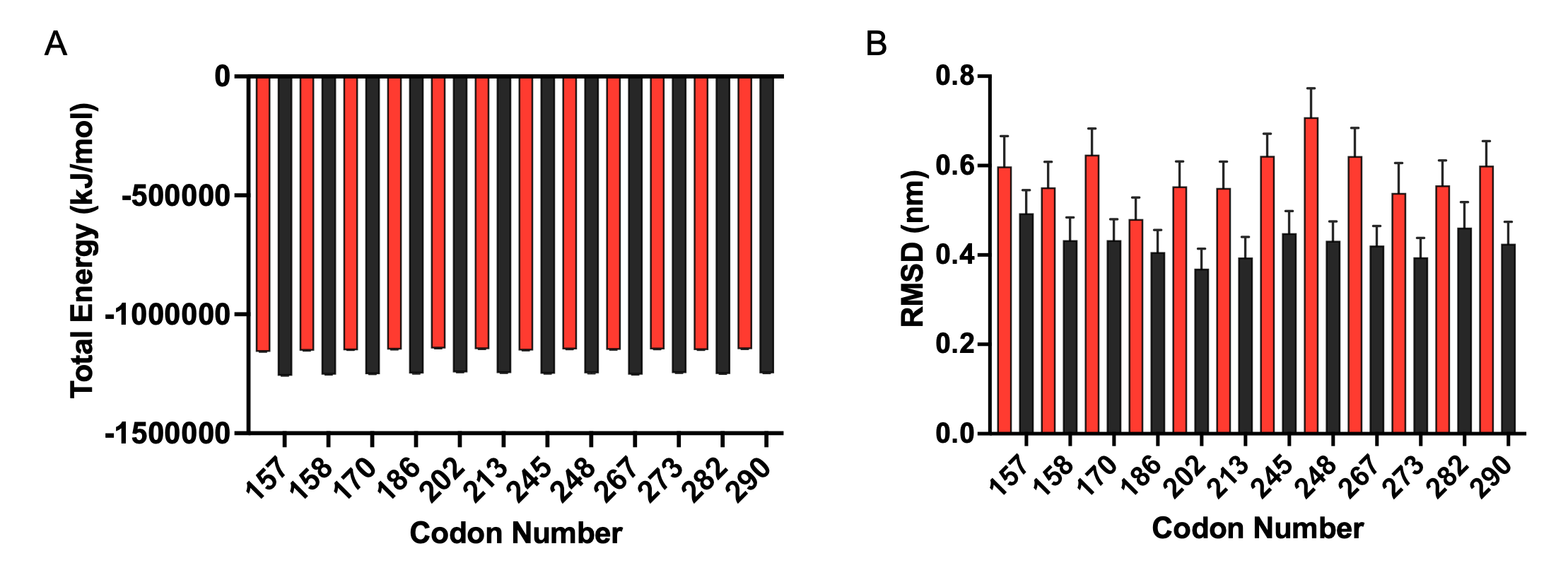
*

*Supplementary Figure S2: Conformational stability and flexibility of adducted and control Meth duplexes. Total energy (****A****) in KJ/mol and RMSD values (****B****) in nm for control sequences (black) and adducted sequences (red); error bars show ± standard deviation.*

*
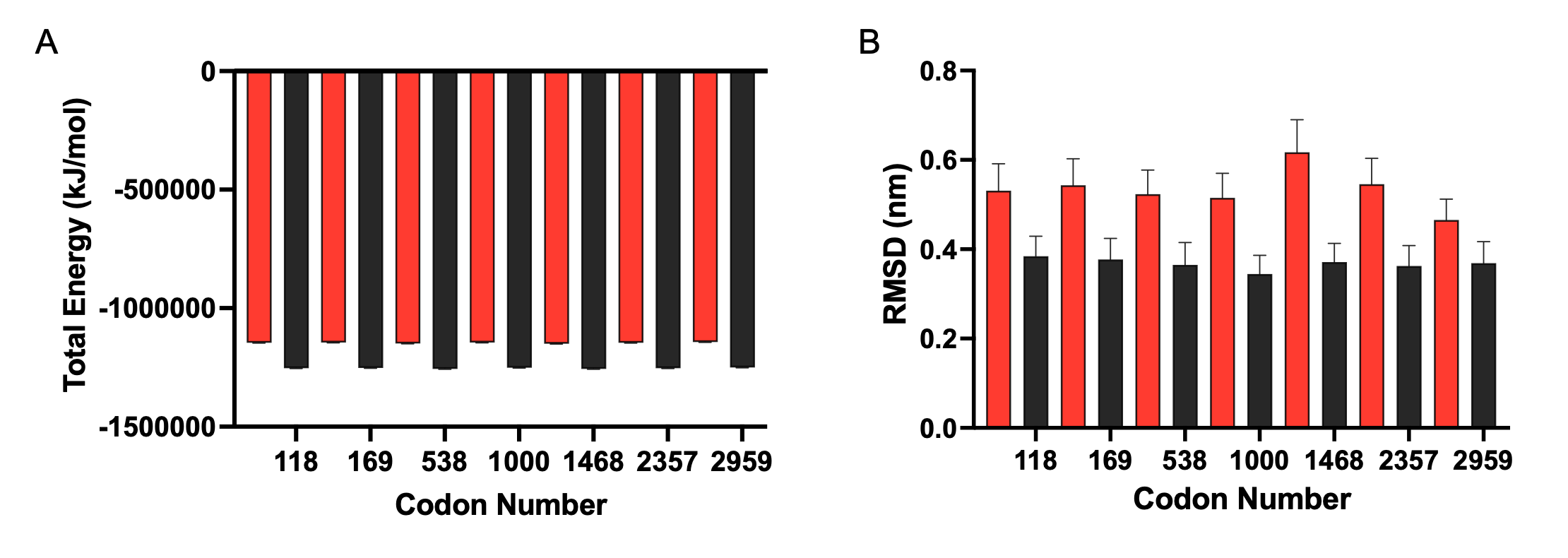
*

*Supplementary Figure S3: Conformational stability and flexibility of adducted and control lacZ duplexes. Total energy (****A****) in KJ/mol and RMSD values (****B****) in nm for control sequences (black) and adducted sequences (red); error bars show ± standard deviation.*

*
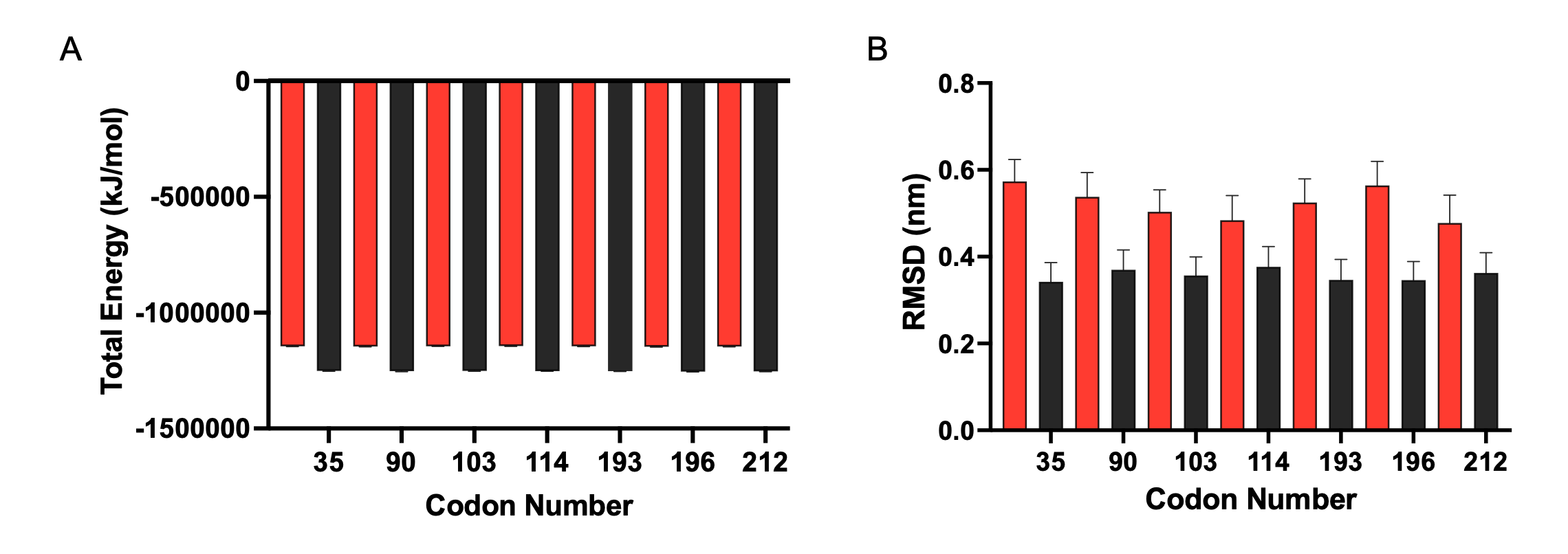
*

*Supplementary Figure S4: Conformational stability and flexibility of adducted and control cII duplexes. Total energy (****A****) in KJ/mol and RMSD values (****B****) in nm for control sequences (black) and adducted sequences (red); error bars show +/− standard deviation.*

*
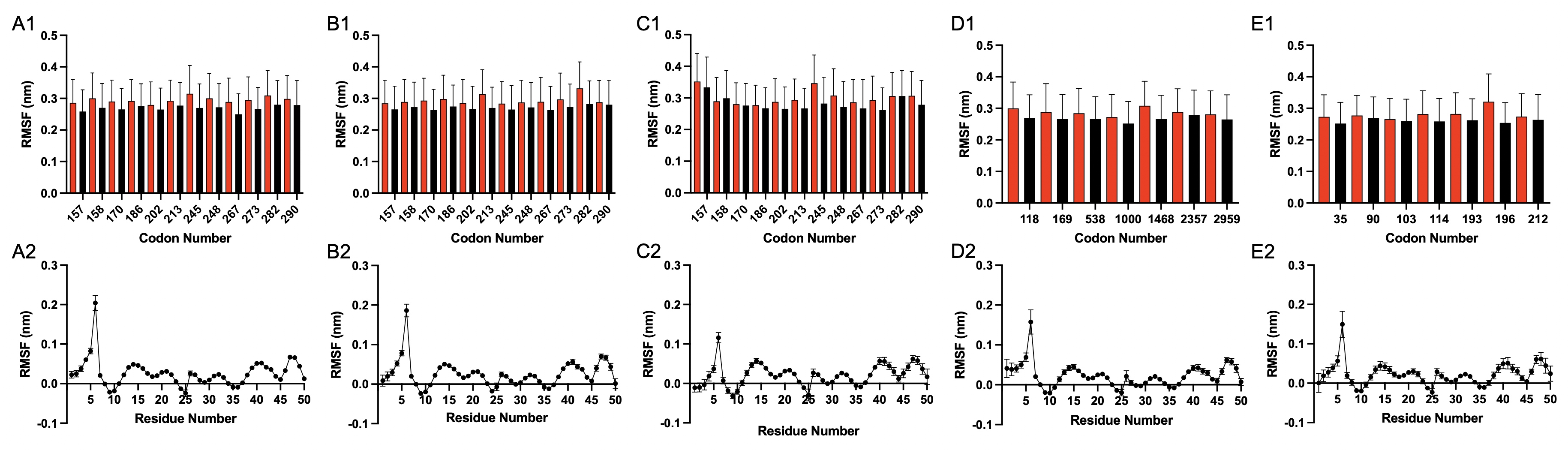
*

*Supplementary Figure S5: Conformational flexibility of adducted and control duplexes. A1-E1, mean whole structure RMSF values in nm for control sequences (black) and adducted sequences (red); error bars show ± standard deviation. A2-E2, context dependent depiction of mean residue wise RMSF in response to BPDE binding. Duplex response was calculated as (Mean RMSF_Adducted duplexes_ – Mean RMSF_Control Duplexes_) and averaged across each dataset; error bars show ± standard error of the mean. All data is presented for datasets TP53 (1), Val (2), Meth (3) lacZ (4), and cII (5).*

*Supplementary* *Table S1:* TP53*DNA sequences used for molecular dynamics simulations (adducted guanine underlined). Sequences were determined to be hotspots in cancers based on their prevalence in the COSMIC database. Difference between GC content of hotspot and non-hotspot sites (base pairs 2-12) was statistically significant (P < 0.005).*

| Codon | Sequence | Hotspot Status (across cancers) | No. observed G:C>T:A in lung cancer | GC Content (%) |
| --- | --- | --- | --- | --- |
| 157 | ACCCGC*GTCCGCGCCATGGCCATCT | Lung | 120 | 72.0 |
| 158 | GCGTCC*GCGCCATGGCCATCTACA | Lung | 107 | 66.7 |
| 245 | ATGGGC*GGCATGAACCGGAGGCCCA | Many | 108 | 68.0 |
| 248 | TGAACC*GGAGGCCCATCCTCACCAT | Many | 75 | 60.0 |
| 273 | AGGTGC*GTGTTTGTGCCTGTCCTGG | Many | 134 | 60.0 |
| 282* | GAGACC*GGCGCACAGAGGAAGAGAA | Many | 1 | 60.0 |
| 170 | CATGAC*GGAGGTTGTGAGGCGCTGC | None | 0 | 64.0 |
| 186 | GATAGC*GATGGTCTGGCCCCTCCTC | None | 0 | 64.0 |
| 202 | ATTTGC*GTGTGGAGTATTTGGATGA | None | 0 | 40.0 |
| 213 | CTTTTC*GACATAGTGTGGTGGTGCC | Breast | 11 | 52.0 |
| 267 | TGGGAC*GGAACAGCTTTGAGGTGCG | None | 2 | 60.0 |
| 290 | ATCTCC*GCAAGAAAGGGGAGCCTCA | None | 0 | 56.0 |

*Sequences displaying majority substitutions distinct from G:C>T:A.

*Supplementary* *Table S2:* cII*DNA sequences used for molecular dynamics simulations (adducted guanine underlined). Sequences were determined to be hotspots in cancers based on the mutational frequency of the*cII*transgene in embryonic mouse fibroblasts upon exposure to BPDE. Difference between GC content of hotspot and non-hotspot sites (base pairs 2-12) was not statistically significant (P > 0.05).*

| Codon | Sequence | Hotspot Status | GC Content (%) |
| --- | --- | --- | --- |
| 35 | CTCTAC*GAATCGAGAGTGCGTTGCT | Hotspot | 52.0 |
| 103 | GTGGGC*GTTGATAAGTCGCAGATCA | Hotspot | 52.0 |
| 193 | GTTGAC*GACGACATGGCTCGATTGG | Hotspot | 56.0 |
| 196 | GACGAC*GACATGGCTCGATTGGCGC | Hotspot | 64.0 |
| 212 | TGTCGC*GCCAATCGAGCCATGTCGT | Hotspot | 60.0 |
| 90 | GACAGC*GGAAGCTGTGGGCGTTGAT | Non-Hotspot | 60.0 |
| 114 | TAAGTC*GCAGATCAGCAGGTGGAAG | Non-Hotspot | 52.0 |

*Supplementary Table S3:* lacZ*DNA sequences used for molecular dynamics simulations (adducted guanine underlined). Sequences were determined to be hotspots in cancers based on their mutational frequency by high throughput next generation sequencing. Difference between GC content of hotspot and non-hotspot sites (base pairs 2-12) was statistically significant (P < 0.005).*

| Codon | Sequence | Hotspot Status | No. observed G:C>T:A upon BPDE exposure | GC Content (%) |
| --- | --- | --- | --- | --- |
| 169 | AATGGC*GAATGGCGCTTTGCCTGGT | Hotspot | 21 | 56.0 |
| 538 | CGCGCC*GGAGAAAACCGCCTCGCGG | Hotspot | 16 | 76.0 |
| 1000 | TTCCGC*GAGGTGCGGATTGAAAATG | Hotspot | 36 | 52.0 |
| 2468 | GGCGGC*GGAGCCGACACCACGGCCA | Hotspot | 17 | 80.0 |
| 2357 | TCACCC*GTGCACCGCTGGATAACGA | Hotspot | 26 | 60.0 |
| 118 | AATAGC*GAAGAGGCCCGCACCGATC | Non-Hotspot | 3 | 60.0 |
| 2959 | AATATC*GACGGTTTCCATATGGGGA | Non-Hotspot | 3 | 44.0 |

*Supplementary Table S4: Helical parameters and their abbreviations.*

| Parameter | Abbreviation | Description |
| --- | --- | --- |
| Base Pair Step Parameters | | |
| Twist/Rise | tw/re | Rotation and Translation of the base pairs about an axis which is perpendicular to the plan of the base step |
| Roll/Slide | rl/se | Rotation and Translation of the base pair about an axis which is the long axis of the base step |
| Tilt/Shift | tt/st | Rotation and Translation of the base pair about an axis which is the short axis of the base step |
| Base Pair Parameters | | |
| Opening/Stagger | og/sg | Rotation and Translation of the bases about an axis which is perpendicular to the plane of the base pair |
| Propeller/Stretch | pl/sh | Rotation and Translation of the bases about an axis which is the long axis of the base pair |
| Buckle/Shear | be/sr | Rotation and Translation of the bases about an axis which is the short axis of the base pair |
| Axis-Displacement Parameters | | |
| X and Y Displacement | xp/yp | Displacement of the bases in the x or y direction |
| Axis-bend | ad | Distance measure of the axis-bend |
| Incline/Tip | in/tp | Angle movement across the x and y axis |

*Supplementary Table S5: Random Forest classification performance with and without the exclusion of simulation time from training data. The metrics include accuracy, precision, recall, and F1-score, with and without the exclusion of 10% (9 ns) of the original simulation time from the TP53 training dataset. The model was then deployed on the original Val dataset, with each value depicting mean scores across 10 distinct training and test splits.*

| Sample Period | Accuracy | Precision | f1 | recall |
| --- | --- | --- | --- | --- |
| 10 – 100 ns | 0.986 | 0.987 | 0.986 | 0.986 |
| 10 – 91 ns | 0.983 | 0.985 | 0.983 | 0.983 |
